# Bilateral cochlear implantation in the ferret: A novel animal model for behavioral studies

**DOI:** 10.1016/j.jneumeth.2010.05.014

**Published:** 2010-07-15

**Authors:** Douglas E.H. Hartley, Tara Vongpaisal, Jin Xu, Robert K. Shepherd, Andrew J. King, Amal Isaiah

**Affiliations:** a Department of Physiology, Anatomy & Genetics, Sherrington Building, Parks Road, Oxford OX1 3PT, UK; b The Bionic Ear Institute, 384-388 Albert St., East Melbourne, VIC 3002, Australia

**Keywords:** Cochlear implant, Ferret, Binaural hearing, Bilateral cochlear implantation, Deafness, Neural prosthesis, Hearing loss, Spatial hearing

## Abstract

Bilateral cochlear implantation has recently been introduced with the aim of improving both speech perception in background noise and sound localization. Although evidence suggests that binaural perception is possible with two cochlear implants, results in humans are variable. To explore potential contributing factors to these variable outcomes, we have developed a behavioral animal model of bilateral cochlear implantation in a novel species, the ferret. Although ferrets are ideally suited to psychophysical and physiological assessments of binaural hearing, cochlear implantation has not been previously described in this species. This paper describes the techniques of deafening with aminoglycoside administration, surgical implantation of an intracochlear array and chronic intracochlear electrical stimulation with monitoring for electrode integrity and efficacy of stimulation. Experiments have been presented elsewhere to show that the model can be used to study behavioral and electrophysiological measures of binaural hearing in chronically implanted animals. This paper demonstrates that cochlear implantation and chronic intracochlear electrical stimulation are both safe and effective in ferrets, opening up the possibility of using this model to study potential protective effects of bilateral cochlear implantation on the developing central auditory pathway. Since ferrets can be used to assess psychophysical and physiological aspects of hearing along with the structure of the auditory pathway in the same animals, we anticipate that this model will help develop novel neuroprosthetic therapies for use in humans.

## 1 Introduction

Approximately 190,000 severe-profoundly deaf individuals worldwide have had their hearing partially restored through cochlear implantation (P. Seligman; personal communication). Animal models have been developed to maximize the benefits of cochlear implantation in humans, whilst using the cochlear implant (CI) as a research tool to study the mechanisms underlying auditory perception. These models have been used effectively to study the effects of acute and chronic unilateral intracochlear electrical stimulation in a variety of species, including the mouse (e.g. Steel and Bock, 1984), rat (e.g. Lu et al., 2005; Millard and Shepherd, 2007), gerbil (e.g. Ryan et al., 1990), guinea pig (e.g. Miller et al., 1983), cat (e.g. Beitel et al., 2000; Klinke et al., 1999; Leake et al., 1991; Ryugo et al., 2005; Smith et al., 1994; Snyder et al., 1995; Vollmer et al., 2001; Xu et al., 1997) and primates (e.g. Pfingst et al., 1979).

In contrast to unilateral CIs, the literature describing bilateral CIs in experimental animals is relatively scant. Recent studies in deaf animals have shown that neurons are sensitive to binaural intracochlear electrical stimulation throughout the IC (Smith and Delgutte, 2007, 2008) and auditory cortex (Hartley et al., 2008; Kral et al., 2009). However these models have not studied behavioral aspects of chronic bilateral intracochlear electrical stimulation. Moreover, most studies of cochlear implantation in animals have involved stimulation of intracochlear electrodes under direct computer control. Although these experiments are conducive to more exact stimulus control, experiments conducted within the free-field create an acoustic environment that is more akin to real-world listening situations. Such free-field studies are needed to supplement the growing body of data collected under conditions of direct intracochlear stimulation. Therefore, we have developed the first model of bilateral cochlear implantation that is suitable for behavioral studies of hearing within a free-field environment, to investigate the effects of chronic bilateral cochlear implantation on the developing brain.

Fitting two cochlear implant systems to most small mammalian species would preclude them from performing a behavioral task within a free-field acoustic environment, because of the additional weight the animal would be required to wear. Ferrets (*Mustela putorius*) are capable of bearing this weight, although cochlear implantation has never been previously described in this species. Whilst ferrets are inexpensive, relative to primates and cats, they are highly suited to behavioral assessments of sensory function. Subsequently, ferrets have been used extensively in auditory research to study the organization (Bizley et al., 2005; Fishbach et al., 2003; Kelly and Judge, 1994; King, 1993; Mrsic-Flogel et al., 2006; Nelken et al., 2008, 2004; Pallas et al., 1990; Phillips et al., 1988; Roe et al., 1992; Shamma et al., 1993), development (Gao et al., 1999, 2000; Gao and Pallas, 1999; Harper and Wallace, 1995; King, 1993; Mrsic-Flogel et al., 2003, 2006; Pallas et al., 1999; Sur et al., 1988; Sur and Leamey, 2001; von Melchner et al., 2000) and plasticity (Elhilali et al., 2007; Fritz et al., 2005a,b, 2003, 2007a,b; King et al., 2007, 2001, 2000; Schnupp et al., 2006; Shechter and Depireux, 2007; Sur and Leamey, 2001) of the central auditory pathway.

Since they are born deaf and do not begin to hear until about a month after birth (Moore and Hine, 1992), manipulations of the acoustic environment and peripheral hearing in ferrets have generated fascinating insights into the role of early experience on central auditory function in this species (Dahmen and King, 2007; Hartley and King, 2010; King et al., 2001, 2000). Following the advent of unilateral and, more recently, bilateral cochlear implantation in children, interest in the role of sensory experience on the developing brain has never been greater (e.g. de Villers-Sidani et al., 2008; Fallon et al., 2009; Leake et al., 2008; Klinke et al., 1999; Kral et al., 2006; Moore and Shannon, 2009; Ohl and Scheich, 2005; Ryugo et al., 2005, 2010; Snyder et al., 1995, 2000; Smith and Delgutte, 2008; Vollmer et al., 1999, 2005; Weinberger and Bakin, 1998; Zhou and Merzenich, 2009).

In this manuscript, we present the results of the first case series of chronic bilateral cochlear implantation in a non-human species to demonstrate the safety and efficacy of our animal model. This novel model can be used to study the effects of chronic intra-cochlear electrical stimulation on the development of the structure and function of the auditory pathway, from free-field behavioral measures of spatial hearing to cochlear morphology. Ultimately we hope that the model will facilitate the development and validation of new technologies and techniques aimed at improving neuroprosthetic devices in humans.

## 2 Methods

### 2.1 Animals

Twenty-seven adult pigmented ferrets (*Mustela putorius*) were used in these studies and all animal procedures were approved by a local ethics committee and licensed by the UK home office (Table 1).

Initially, we examined the effectiveness of our chronic bilateral cochlear implantation technique in the ferret. Specifically, electrode impedances and electrically evoked auditory compound action potentials (ECAPs) were measured over a period of three post-operative months following bilateral CI surgeries. Four adult ferrets were deafened following administration of neomycin (see section below) and chronically implanted with intra-cochlear electrode arrays in both ears. Otoscopy was performed prior to cochlear implantation to ensure that both ears were disease free. At the time of CI surgery, the animals were, on average, 314 days old (range 135–484 days old). Profound bilateral sensorineural hearing loss was confirmed in all animals, as evidenced by no ABRs to clicks presented at >95 dB SPL. Apart from transient ataxia in one ferret that had received intrascalar administration of neomycin, which was attributed to vestibular dysfunction, all animals recovered from bilateral cochlear implantation without complication. The implants were monitored for periods ranging from 75 to 120 days.

Additional studies were conducted in adult ferrets to assess our ability to match the depth of implantation across the two ears (*n* = 2), and to investigate potential binaural cues that would be provided by externally worn speech processors (*n* = 21). These studies are described in more detail below.

### 2.2 Deafening technique

Animals were deafened with either systemic (*n* = 2) or bilateral intrascalar (*n* = 2) aminoglycoside administration. The systemic deafening technique permitted the time of cochlear implantation to be separated from the age at onset of hearing loss. Conversely, for animals deafened via direct cochlear infusion, the age at onset of hearing loss was synchronous with the age at implantation. For systemic aminoglycoside administration, subcutaneous injections of neomycin sulphate (Sigma–Aldrich, Poole, Dorset, UK) were administered at a dose of 30–50 mg kg^−1^ day^−1^ for a maximum of 21 days (Leake and Hradek, 1988). Approximately 2 weeks after commencing the ototoxic treatment, the efficacy of the deafening procedure was assessed by click-evoked auditory brainstem responses (ABRs) under sedation provided by intramuscular administration of medetomidine hydrochloride (Domitor; 0.08 mg/kg; Pfizer, Sandwich, UK). Normally hearing animals have strong responses to clicks and tone pips, presented over a wide range of levels (Moore and Hine, 1992). In neomycin treated animals, if click-evoked ABRs were recorded, daily injections were continued. Injections were discontinued when profound bilateral hearing loss was confirmed through the absence of an ABR to acoustic clicks presented at >95 dB SPL. During the deafening procedure, animals were observed for signs of nephrotoxicity. In addition to monitoring systemic health (e.g. weight, food and water intake), urine was examined daily for proteinuria, haematuria or glycosuria using reagent strips (Uristix, Bayer, UK) and for specific gravity using a handheld clinical refractometer (Atago, Japan). No abnormalities were detected in either animal.

Intrascalar administration of neomycin was performed at the time of cochlear implantation in two other chronically implanted ferrets. The anesthetic regime and surgical approach to the cochlea are described in detail below. After the round window was opened with a 23-gauge hypodermic needle, the scala tympani was gently irrigated with approximately 1 ml of neomycin sulphate (10 mg/ml in normal saline) over a period of five minutes (Hardie and Shepherd, 1999). Subsequently, the intracochlear electrode array was implanted and fixed using the technique described below. Again, ABRs were measured to confirm deafness.

### 2.3 Electrode design

**Fig. 1:**
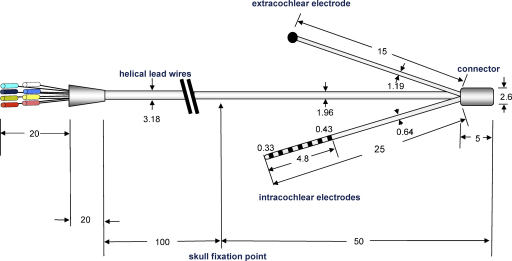
Diagram of an electrode assembly suitable for bilateral implantation in the ferret (all dimensions in mm). The intracochlear portion of the array consists of 7 platinum ring electrodes with an inter-electrode separation of ∼0.4 mm. At the skull fixation point, a wide piece of Dacron mesh was fixed to the lead wire to protect it from the titanium skull fixation clip.

A custom-made electrode assembly suitable for bilateral cochlear implantation in the ferret was developed, based on previous implants used in the rat (Lu et al., 2005), guinea pig (Shepherd and Xu, 2002) and cat (Xu et al., 1997). The electrode assembly consists of an intracochlear electrode array consisting of seven platinum ring electrodes (0.33–0.43 mm in diameter with an inter-electrode separation of ∼0.4 mm), an extracochlear platinum ball electrode, a connector and lead wires (Fig. 1). The 7th electrode from the tip was useful for accurately judging the depth of implantation via the round window and for ensuring an even depth of implantation across the two ears. Teflon-insulated platinum-iridium (90/10) wire, 25 μm diameter, was used to connect the active electrode rings to Teflon-insulated stainless-steel lead wires, embedded in a silicone carrier.

### 2.4 Surgical approach

Anesthesia was induced by intramuscular administration of medetomidine hydrochloride (Domitor; 0.08 mg/kg; Pfizer, Sandwich, UK). At the time of induction, an intramuscular injection of atropine sulphate (0.1 mg/kg, C-Vet Veterinary Products, Leyland, UK) was given to reduce the risk of bradyarrhythmias and to dry airway secretions. At the time of induction, subcutaneous injections were given of (i) buprenorphine (Vetergesic; 0.05 mg/kg; Alstoe Animal Health) and meloxicam (Metacam; 0.2 mg/kg; Boehringer-Ingelheim) for analgesia, and (ii) co-amoxiclav (Synulox RTU; 20 mg/kg; Pfizer, USA) for antibiotic prophylaxis. Following induction, a 24 gauge cannula was inserted into the cephalic vein. Anesthesia was maintained with a continuous infusion of propofol (PropoFlo; 1 mg/kg/h; Abbott Animal Health) and ketamine (Ketaset; 5 mg/kg/h; Fort Dodge Animal Health, Southampton, UK) in 5% glucose/saline solution. Under alternative anesthetic regimes in this species, such as isoflurane, the authors frequently noted severe bradyarrhythmias that were not abolished with atropine during bilateral CI surgery. Similar vagal reflexes have been reported during a variety of surgical manipulations in the ferret, associated particularly with isoflurane anesthesia (Johnson-Delaney, 2005). These arrhythmias were not observed when anesthesia was maintained using a propofol and ketamine infusion.

Animals were intubated with a 3-mm internal diameter, uncuffed, endotracheal tube (Portex^®^, Smiths Medical International Ltd., Hythe, Kent, UK) inserted under direct laryngoscopy. The appropriate depth of endotracheal insertion was estimated by measuring the distance between the animal's incisors and a mid-point between the scapulae and the external occipital protuberance. Subsequently, animals were ventilated with an oxygen/air mix, and body temperature, end-tidal CO_2_, and electrocardiogram were monitored throughout the surgical procedure. Ocular lubrication (Viscotears; Novartis Pharms, UK) was applied during the procedure to protect the eyes.

The surgical approach was developed through cadaver dissections. During the surgical procedure the anaesthetized animal was positioned in a lateral recumbent position with a small shoulder bolster placed under the head to laterally flex the neck. Under a sterile surgical technique, a post-auricular skin incision was made and the round window was exposed by drilling a hole in the bony tympanic bulla. With the aid of an operating microscope (Carl Zeiss Meditec Inc.), the round window was incised using a 23-gauge hypodermic needle, being careful to minimize leak of perilymphatic fluid, and sodium hyaluronate (Healon^®^, Advanced Medical Optics, USA) was injected around the incision to provide lubrication during insertion of the electrode array. Subsequently, the electrode array was carefully inserted through the round window incision into the scala tympani to a depth of approximately 7.5 mm from the round window niche. A small piece of fascia was placed around the array to seal the hole in the round window and minimize the potential of perilymphatic fluid leak. The array was attached to the leadwire via a connector that was fixed within the bulla using antibiotic-impregnated bone cement (DePuy CMW Gentamicin, DePuy International Ltd., UK) and a Dacron mesh tie. The hole in the bulla was sealed with more bone cement, through which the lead wire passed. The lead wire was buried beneath the temporalis muscle and fixed to the skull at two points within the temporal fossa using a custom-designed titanium plate and screw (United Titanium Inc., Wooster, Ohio). The extracochlear electrode was positioned beneath the temporalis muscle. During bilateral cochlear implantation the procedure was repeated on the contralateral side. Lead wires exited the skin between the scapulae.

### 2.5 Depth of implantation

**Fig. 2:**
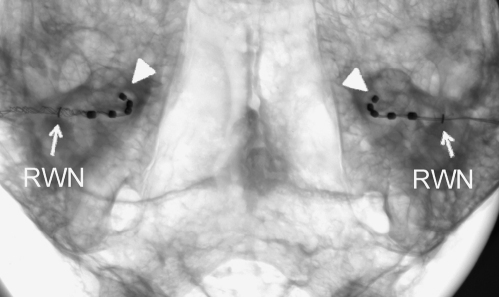
High resolution micro-focus radiograph in anterior–posterior view of a ferret skull showing bilateral intracochlear arrays inserted to an even depth into the basal turn of both cochleas relative to the round window niche (RWN) marked with a fine wire. The apical electrode in the electrode array is marked with an arrowhead.

Human psychophysical studies suggest that mismatching the cochlear position of electrical stimulation between the ears by greater than 2 mm can degrade binaural sensitivity (van Hoesel, 2004). To assess whether an even depth of insertion of the electrode arrays could be achieved between the ears, analysis of micro-focus X-rays was used (Xu and Cowan, 2005). In adult ferret cadavers (*n* = 2), a small radio-opaque marker wire was fixed to the round window niche (Fig. 2). An intracochlear electrode array was then inserted into the scala tympani using the same surgical approach as that used for live cochlear implant surgery. To facilitate an even depth of electrode insertion in both ears, the basal most electrode (7th electrode) was positioned at the level of the round window niche. Subsequently, micro-focus X-rays were analyzed to measure the distance between the round window niche and the apical electrode in each ear (Fig. 2).

### 2.6 Effectiveness of implantation

**Fig. 3:**
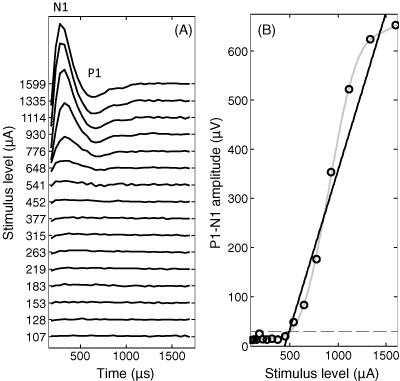
(A) Representative ECAP waveforms from a ferret at the stimulus amplitudes indicated. (B) P1–N1 amplitude plotted as a function of stimulus level for the same recording with best-fitting linear regression (solid black line). P1–N1 amplitudes of less than 30 μV (dashed line) were considered likely to be within the noise floor. Therefore they were excluded from threshold estimation analysis.

Daily electrode impedance measurements and weekly electrically evoked compound action potentials (ECAP; Nagel, 1974) were recorded in awake animals using dedicated software (Custom Sound™ EP, Cochlear Ltd.) by connecting their lead wires to a programming interface (Nucleus Freedom sound processor and programming pod, Cochlear Ltd.). Impedance measurements were collected for each of the intracochlear electrodes referenced against all other electrodes in common-ground (CG) configuration, and against the extracochlear platinum ball electrode in monopolar (MP) configuration. For ECAP recordings each intracochlear electrode was stimulated in MP configuration using pairs of charge balanced biphasic current pulses (25 μs/phase), whilst an adjacent electrode along the array was used as the recording electrode referenced against the extracochlear electrode. Stimulus artifact was removed from the ECAP recordings using a modified version of the forward-masking paradigm (Brown et al., 1990; de Sauvage et al., 1983), which was implemented by the Custom Sound™ EP software. Recordings were analyzed offline by fitting a linear regression to the P1–N1 amplitude growth function (Fig. 3). As previously described by Dillier et al. (2002), the ECAP threshold was defined as the stimulus value at the zero crossing of the extrapolated P1–N1 amplitude function.

### 2.7 Chronic stimulation

**Fig. 4:**
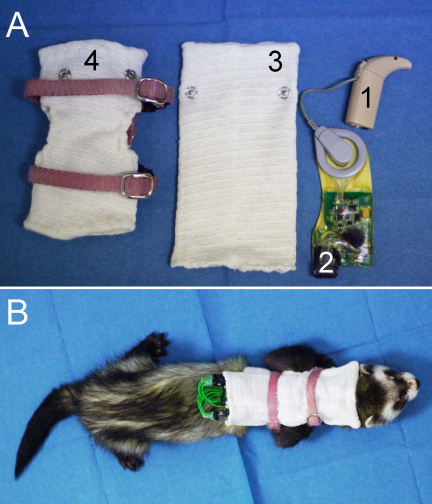
(A) Nucleus ESPrit 3G speech processor (1) attached to a modified Nucleus Cochlear implant CI24RE emulator (2). Pockets were incorporated within the neckline of a detachable ‘backpack’ (3) that was attached to a jacket made from a ferret harness and elasticated tubular bandage (4). (B) Ferrets carried their cochlear implants within the jacket that held the microphone of the left and right speech processor immediately posterior to the ipsilateral pinna and enabled animals to carry on with their normal activities during chronic stimulation.

Chronic intracochlear electrical stimulation was commenced in all animals approximately 2 weeks after CI surgery. Animals were stimulated on average 10 h per day, 7 days per week to imitate the usage of CI speech processors amongst clinical populations. The apical six electrodes in each ear were stimulated by coupling the percutaneous leadwire to a modified stimulator-receiver (Nucleus Cochlear implant CI24RE emulator, Cochlear, Englewood, CO; Fig. 4A; weight ∼15 g) and Nucleus ESPrit 3G speech processor (Cochlear, Englewood, CO; Fig. 4A; weight ∼15 g), using similar techniques to those previously described by Fallon et al. (2009) in the cat. Since each animal had bilateral CIs, the combined weight of the receiver-stimulators and speech processors was approximately 60 g. Speech processors were programmed to deliver biphasic current pulses (37 μs per phase) at 900 pulses per second per electrode using a monopolar electrode configuration. The upper and lower stimulus levels were initially set to 0 and 6.3 dB below the ECAP threshold. Subsequently, stimulation levels were assessed using behavioural observations, including the head orienting response. These assessments were used to fine-tune the lower and upper levels of stimulation to the estimated perceptual threshold and comfort levels, respectively. The animals carried their CIs within a backpack so that they were able to assume their normal activities whilst being stimulated within an acoustic environment enriched with animal and human vocalizations and other sounds associated with the normal day-to-day running of the facility in which they were housed (Fig. 4B).

### 2.8 Preservation of binaural cues during chronic stimulation

Interaural level differences (ILDs) and interaural time delays (ITDs) arising from the binaural disparity of a sound arriving at the two ears are the predominant cues for localization of a sound in the horizontal plane (azimuth). During the development of this model it was important to ensure that chronically stimulated animals with bilateral CIs were provided with behaviourally relevant binaural cues. Subsequently, a jacket was developed using a ferret harness and elasticated tubular bandage (‘Tubigrip’ size D, Mölnlycke Health Care, Sweden) that animals wore both to support the speech processors and modified stimulator receivers and to protect the percutaneous lead wires (Fig. 4A). Specifically, ‘pockets’ were incorporated within the neckline of a detachable ‘backpack’ that held the microphone of the left and right speech processor immediately posterior to the ipsilateral pinna. The elastic tubular material allowed the microphones to move laterally or vertically with the ears as the animal changed the position of its head, whilst the harness sewn into the jacket prohibited caudal, rostral or circumferential movement of the speech processors in relation to the animal's body.

**Fig. 5:**
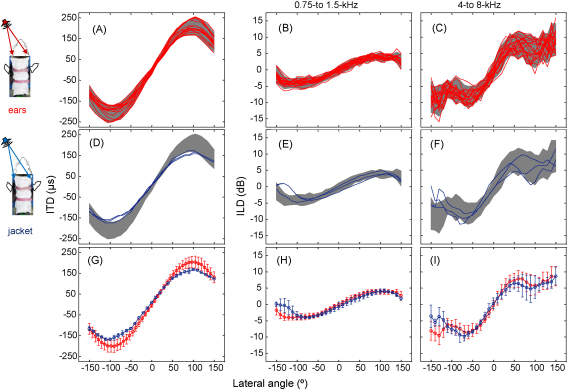
(A) ITDs measured from the external ear canals of sixteen adult ferrets (red lines) plotted as a function of lateral angle. Here, a negative lateral angle denotes a position to the animal's left. (B and C) ILDs measured from the external ear canals of adult ferrets (red lines; *n* = 16) for sounds filtered between 0.75 and 1.5 kHz (B) and 4 and 8 kHz (C) are plotted as a function of lateral angle. The confidence interval for the group mean is represented by the grey shaded area (5–95%). ITDs (D) and ILDs separated by frequency (E and F) measured from the “pockets” of a custom-made jacket worn by three adult ferrets are plotted in blue (mean + s.d.) as a function of lateral angle. For comparison, the confidence intervals for the external ear canal measurements are re-plotted in grey. Mean (+s.d.) ITDs (G) and ILDs separated by frequency (H and I) are shown for the external ear canal measurements in red and for the jacket pockets in blue.

To ensure the jacket preserved behaviourally relevant binaural cues, acoustical measurements were made using the microphones attached to probe tubes placed in the neckline ‘pockets’ of three anaesthetized animals wearing the jacket. These were compared with recordings from the external ear canals of sixteen normal adult ferrets (Fig. 5) under anesthesia taken from previous studies from our laboratory (Mrsic-Flogel et al., 2001; Schnupp et al., 2003). In those animals, polythene tubes were inserted (∼length 2 cm, outside and inside diameters 1.52 and 0.86 mm, respectively) into the posterior aspect of the external ear canal, around the junction of the bony and cartilaginous portions, through a small post-auricular incision such that the tube opened into the meatus without protrusion. The animal was placed in the centre of a robotic hoop (65 cm radius and 0° elevation) within an anechoic chamber whilst signals were presented from a speaker (Kef T27) mounted on the robotic hoop. The hoop moved the speaker automatically in 10° intervals to present signals from a range of positions (±150° azimuth, where negative values denote positions to the left). Signals were recorded from microphones, preamplified (M-Audio DMP3 dual microphone/instrument preamp, Taiwan) and digitized at a sample rate of 80 kHz. The probe stimuli were 2000-ms bursts of broadband noise and 512 point Golay code pairs (sampled at 80 kHz and antialias filtered at 30 kHz; Zhou et al., 1992). Stimuli were presented at a level of 95 dB SPL at each speaker whilst speaker output was re-recorded from microphones (Sennheiser microphone capsules KE-4-211-2) attached to probe tubes inserted in the external ear canals and the jacket ‘pockets’. A previous study in our laboratory showed that interaural time delays (ITDs) recorded from the external ear canals of ferrets are largely independent of sound frequency (Schnupp et al., 2003). Therefore, in the current study, ITDs were extracted from the unfiltered recorded impulse response functions using cross correlation. In contrast, interaural level differences (ILDs) vary significantly across frequency, therefore recordings were bandpass filtered prior to calculating ILDs by subtracting the root-mean square energy at each ear.

**Fig. 6:**
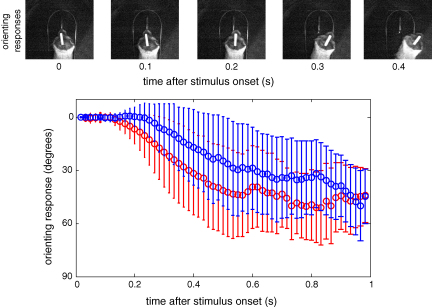
Sound evoked head and jacket orienting responses. The upper panels show the orienting movements of a ferret performing a free-field sound localization task recorded from an infrared camera (60 frames s^−1^) using a reflective strip attached to the animal's head. The lower panel shows how the horizontal angle of the animal's head (red circles; mean + s.d.; *n* = 1110 trials) and jacket (blue circles; mean + s.d.; *n* = 932 trials) changed after a stimulus was presented from the +90° speaker position.

In order to assess how well the pocket microphones replicate the binaural cues available when an animal turns towards a sound source, we measured the orienting responses of a normal-hearing ferret (*n* = 1) that had been trained by positive conditioning to perform a sound detection task, whilst carrying two speech processors and two modified stimulator-receivers within a jacket (Fig. 6). Methods used to measure head orienting responses in the ferret have been described in detail elsewhere (Nodal et al., 2008). Briefly, the task was carried out in a testing arena (70 cm radius) located inside a sound-attenuated chamber. The animal had to stand on a platform at the center of the arena and initiate a trial by licking a centrally positioned waterspout. This ensured that its head was positioned at the centre of the arena and facing straight ahead at the beginning of each trial. The animal was trained to lick the start spout continuously for 0.5–2 s until either (i) a continuous 500 Hz, 80 dB SPL pure tone was presented from a laterally placed loudspeaker (at +90°), or (ii) no stimulus was presented. Water rewards were delivered only if the animal made a correct response by licking (i) the spout associated with the +90° speaker, if a tone was presented, or (ii) a second spout associated with the −90° position, at the perimeter of the arena, if no stimulus was presented. We measured the change in the animal's head orientation following the presentation of the tone by tracking the movement of a self-adhesive reflective strip attached to an area of shaved skin along the midline of the animal's head (Fig. 6, top panel). In separate trials we measured the movement of the animal's jacket by attaching a reflective strip between the jacket pockets. The *x*–*y* coordinates of the reflective strip were registered for 1 s following stimulus onset at a rate of 60 frames per second using an overhead infrared-sensitive camera and video contrast detection device (HVS Image, Harlow, UK). These coordinates were used to derive the angular extent of the orienting response relative to the initial position. Only trials in which the tone was presented were analyzed. As in previous studies (Nodal et al., 2008), the ‘latency of the movement’ was defined as the third frame of three that showed a consecutive movement in the same direction after the stimulus onset. The ‘final bearing’ was calculated as the mean angle from the last three frames recorded during the 1 s over which the coordinates were sampled.

**Fig. 7:**
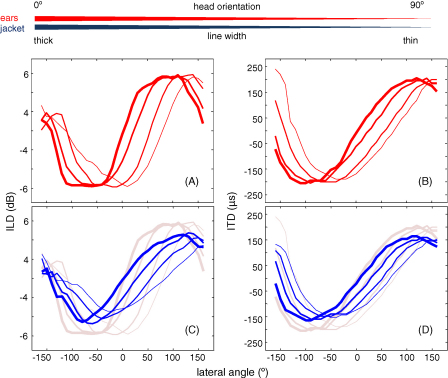
To model the effects of head movement on binaural cues, the animal's head orientation (*n* = 1) was moved within the chamber in increments of 30° from the 0° speaker position towards the +90° speaker position. In each orientation of the animal, (A) ILDs and (B) ITDs were measured from the external ear canals of an adult ferret (red lines) plotted as a function of lateral angle of the sound source. The thickness of the line represents the head position of the animal within the chamber, with decreasing line thickness being associated with head positions further away from the 0° speaker position. (C) ILDs and (D) ITDs measured from microphones positioned within the jacket pockets as a function of lateral speaker position and head position within the chamber. For reference, data recorded from the external ear canals are replotted in gray (C and D).

**Fig. 8:**
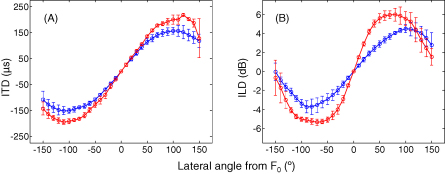
Data from figure are replotted to show variation between successive ILD and ITD measurements within the same animal. (A) ITDs and (B) ILDs (mean + s.d.) measured from the external ear canals (red circles) and jacket pockets (blue circles) of an adult ferret positioned four times within the chamber, plotted as a function of lateral angle away from the zero crossing for each recording (F_0_).

We also wanted to assess how the binaural cues provided by the jacket might change as the animals moved around in their acoustic environment. Fitting probe tubes to the external ear canals of awake, behaving ferrets would be problematic since the tube would be prone to becoming blocked with wax, may change position, cause irritation or become infected. Therefore, in an attempt to model the head orienting response of an animal performing a sound localization task (Fig. 6, top panel), further acoustic recordings were made from the jacket pockets and external ear canals of an animal in 4 different positions (Figs. 7 and 8). Specifically, a fine surgical suture was fixed to the animal's nasal columella prior to positioning the animal in the centre of the speaker loop. The other end of the suture was fixed to the periphery of the chamber, so that the suture and the animal's body were directly in line with the 0° speaker. In this position, acoustic measurements were recorded from both the external ear canals and jacket pockets in an identical way to those described above. In three subsequent recordings, the animal's orientation within the chamber was altered by moving the suture in increments of 30° from the 0° speaker position, towards the +90° degree speaker position. In each orientation of the animal, further measurements were taken from the external ear canals and jacket pockets.

To assess potential noise generated by the jacket placement of the microphones, additional acoustic measurements were taken (i) from a microphone placed inside and outside the jacket pockets, (ii) before and after an animal wore the jacket (F0822).

## 3 Results

### 3.1 Deafening

Profound bilateral sensorineural hearing loss was confirmed in all 4 animals through the absence of ABRs to clicks presented at >95 dB SPL. In animals treated with subcutaneous aminoglycoside administration, the click-evoked ABR was abolished between 15 and 18 days after commencing the neomycin protocol.

### 3.2 Depth of Implantation

Analysis of anterior–posterior X-rays taken in adult ferret cadavers (*n* = 2) suggested that, on average, the mean distance between the round window niche and the apical electrode of an intracochlear array was 7.2 mm (range 7.1–7.3 mm; *n* = 4 ears). Although these results suggest that the depth of implantation of the apical electrode varies by ∼0.2 mm across the ears (Fig. 2), it should be noted that this observation is based upon a limited data set.

### 3.3 Effectiveness of implantation

**Fig. 9:**
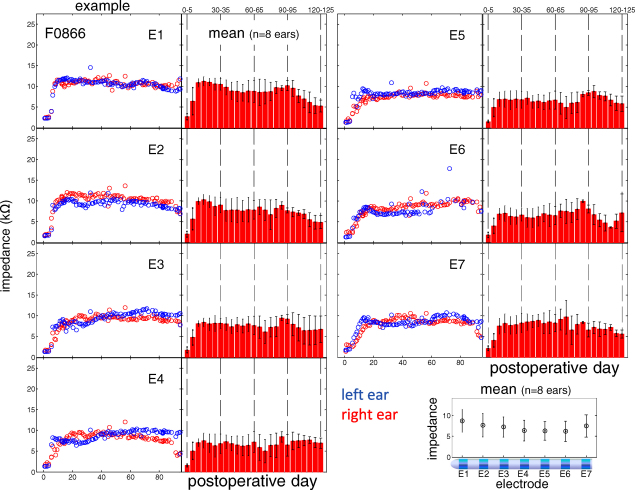
Impedance measurements for (i) one representative animal (F0866; 1st and 3rd columns; measurements from the left and right ears in blue and red, respectively), and (ii) averaged across both ears of all four animals (2nd and 4th columns), plotted as a function of intracochlear electrode position (AE1–AE7) and post-operative day. In the bottom right panel, mean impedance measurements (±s.d.) are shown for each electrode position, for all 8 ears (*n* = 4 animals).

During electrode impedance measurement, each intracochlear electrode position was, in turn, designated the active electrode whilst the indifferent electrode was either (i) the remaining intracochlear electrodes in the ipsilateral ear (common-ground = CG), or (ii) the ipsilateral extracochlear ball electrode (monopolar = MP). Fig. 9 shows impedance measurements in CG for each electrode position (AE1–AE7) in one representative animal (F0866; 1st and 3rd columns) and impedance measurements in CG for each electrode position averaged across both ears of all four animals (Fig. 9, 2nd and 4th columns). Impedance measurements varied across animals, electrode position and over time. Across the electrode array, impedance measurements were highest at the apical electrode position and were lowest in the middle of the array (AE5; Fig. 9, bottom right panel). A repeated measures analysis of variance (ANOVA) revealed a significant main effect for electrode position (*F*_6, 96_ = 37.3, *p* < 0.001), and a significant interaction between animal and electrode position (*F*_6, 96_ = 37.3, *p* < 0.001). On average, electrode impedances were 1.9 kΩ (s.d. 0.7) prior to the 5th post-operative day. These measurements steadily increased to be, on average, 8.5 kΩ (s.d. 2.2) between 16 and 20 days following implantation. Thereafter, impedance measurements remained relatively stable, or slightly decreased in some cases, across different electrode positions (Fig. 9). If the impedance measurement was <1 kΩ, or >20 kΩ, the electrode was no longer used and was described as ‘closed-circuit’ or ‘open-circuit’, respectively.

Impedance measurements for the four animals remained within the desired range (1–20 kΩ) for the majority of electrode positions (94%) over the duration of testing. Impedances remained within this range for all electrodes in both arrays of two of these animals (*n* = 32 electrode positions; 100%) across all measurements. In the remaining two animals, impedance measurements indicated open-circuit for 6 out of 32 electrode positions between the 43rd and 79th post-operative day. For these animals, impedance measurements remained within the desired range for all remaining electrode positions (26 out of 32) for the duration of testing. Modifications to the surgical technique reduced the incidence of open circuit electrodes. These modifications included the use of skull fixation clips and implanting the connector segment of the electrode assembly within the bony mastoid bulla.

**Fig. 10:**
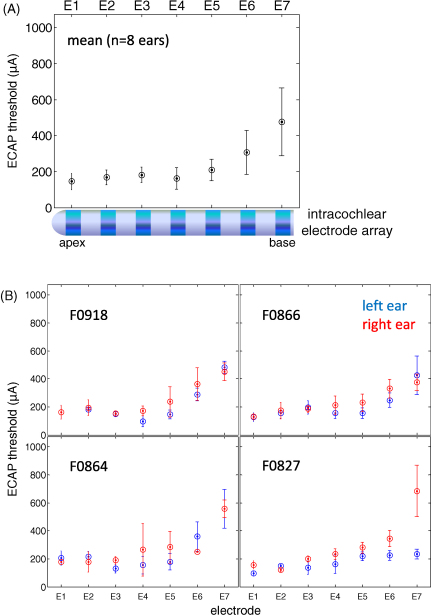
(A) Mean ECAP thresholds across all measurements (+s.d.) plotted as a function of electrode position, for all 8 ears (*n* = 4 animals). (B) Mean ECAP thresholds for the left (blue) and right (red) ears are also shown for individual animals as a function of electrode position.

**Fig. 11:**
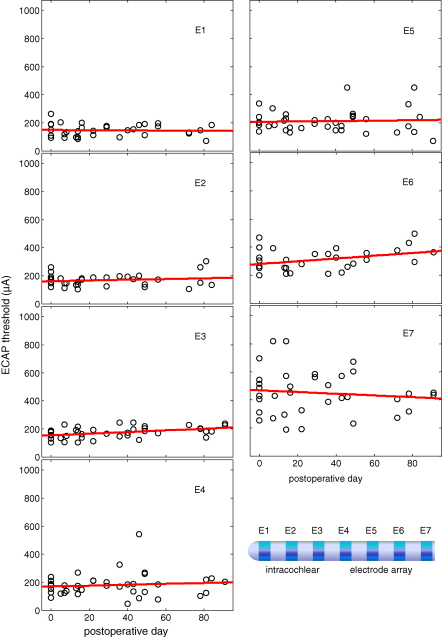
ECAP thresholds plotted for each electrode position as a function of post-operative day with best-fitting linear regressions. Each circle represents one ear of the four animals.

ECAP thresholds were highest for electrode positions towards the basal end of the array (Fig. 10). However, thresholds varied little between animals (Fig. 10) and remained relatively stable over time (Fig. 11). A repeated measures ANOVA revealed a significant main effect for electrode position (*F*_6, 96_ = 37.3, *p* < 0.001), but no significant interaction between animal and electrode position. A post hoc pairwise multiple comparisons procedure, with Bonferroni adjustment for multiple comparisons, indicated that ECAP thresholds were significantly higher for electrode position AE7 and position AE6 compared with all other electrode positions. Fig. 11 shows ECAP thresholds plotted against time for each electrode position, with best fitting linear regression. The mean slope of the linear regressions across all 7 electrode positions was 0.2 (range: −0.7 to 1.1), and was not significantly different from zero (*p* = 0.4), indicating that ECAP thresholds remain constant over time.

### 3.4 Preservation of binaural cues during chronic stimulation

When signals were presented from an array of speaker positions (±150°), overall ITDs and ILDs measured from the external ear canals of 16 normal adult ferrets were comparable to measurements taken from pockets of the custom-made jacket (Fig. 5). Across our cohort of animals, the smallest range of ITDs measured from an animal's external ear canals was ±169 μs, and the largest range of ITDs was ±254 μs (median range = ± 190 μs; *n* = 16; Fig. 5A and G), a reflection of inter-animal differences in head size. The smallest range of ITDs measured from the pockets of a custom-designed jacket worn by an animal was ±163 μs and the largest range of ITDs was ±174 μs (median range = ± 169 μs; *n* = 3; Fig. 5D and G). Therefore, all jacket pocket measurements closely matched data obtained from the external ear canals of animals within our cohort with the smallest range of ITD measurements (Fig. 5A and G). The variability of ITD measurements between animals, and median range of ITDs, were both smaller for jacket pocket, compared with the external ear canal measurements. This may partially reflect the large number of external ear canal measurements in our data set. Furthermore, the range of ITDs provided by the jacket could potentially be modified by varying the distance between the pockets.

When the signal was bandpass filtered from 0.75 to 1.5 kHz, the smallest range of ILDs measured from the external ear canals of an animal was ±3.5 dB, and the largest measured range was ±6.4 dB (median range = ± 4.3 dB; *n* = 16; Fig. 5B and H). When the signal was bandpass filtered from 4 to 8 kHz, the smallest range of ILDs measured from the external ear canals of an animal was ±9.0 dB, and the largest measured range was ±15.9 dB (median range = ± 10.9 dB; *n* = 16; Fig. 5C and I). The median range of ILDs measured from the pockets of the custom-designed jacket was ±4.2 dB (Fig. 5E and H) and ±9.8 dB (Fig. 5F and I) after signals were bandpass filtered from 0.75 to 1.25 kHz and 4 to 8 kHz, respectively. Regardless of whether the measurements were taken from the external ear canals or jacket pockets, the variation in ILD measurements between animals was comparable (Fig. 5H and I).

These measurements therefore show that the ITDs and ILDs provided by the jacket microphones are very similar to those available at the external ears of ferrets with normal hearing. Because the ferrets have to adopt a consistent head position in our auditory localization behavioural task (Nodal et al., 2008), this shows that the implanted ferrets experience binaural cue values that fall within the normal range.

Fig. 6 compares the orienting response measurements taken from the head and from the jacket of a single animal performing a sound detection task. Although the response latency measured from the head was, on average, 117 ms shorter than that from the jacket, the change in position of both the head and the jacket approximated sigmoid functions. Importantly, the mean ‘final bearing’ derived from the head and jacket measurements were almost identical (42.96° and 41.69°, respectively).

Fig. 7 compares ITD and ILD measurements from the external ear canals of a single ferret with measurements taken from the pockets of the jacket, as the animal's head orientation within the chamber was moved from the 0° speaker towards the 90° speaker in 30° increments. As the head orientation changed, incremental shifts were seen in ITD and ILD measurements from both the external ear canals (Fig. 7A and B) and jacket pockets (Fig. 7C and D). For example, ITDs measured from the external ear canals in response to signals presented from the 40° speaker were 102 μs, 41 μs, −46 μs and −87 μs with the head oriented towards the 0°, 30°, 60° and 90° speaker position, respectively. During the same movements of the animal's head, ITDs measured from the jacket pockets in response to signals presented from the same speaker were 82 μs (0° orientation), 26 μs (30° orientation), −26 μs (60° orientation) and −61 μs (90° orientation). Again, as signals were presented from the 40° speaker, unfiltered ILDs measured from i) the external ear canals were 4.4 dB (0° orientation), 2.3 dB (30° orientation), −2.4 dB (60° orientation) and −4.5 dB (90° orientation), and ii) the jacket pockets were 2.5 dB (0° orientation), 0.8 dB (30° orientation), −1.1 dB (60° orientation) and −2.0 dB (90° orientation).

Data presented in Fig. 7 were recorded from the same animal in 4 different orientations within the chamber. To assess the variability between successive external ear canal and jacket pocket measurements, these data were reanalyzed to align the zero crossings of each ITD and ILD recording (Fig. 8). Specifically, for each recording, the lateral speaker angle that that was associated with an ITD or ILD equal to zero was aligned to zero on the *x*-axis (F_0_). The remaining speaker positions for each recording were labeled with respect to the lateral angle they made with the F_0_ position. This analysis shows that the variability between successive ITD and ILD recordings was similar between external ear canal and jacket pocket measurements (Fig. 8A and B).

### 3.5 Noise generated by the jacket placed microphones?

To investigate potential noise generated by the jacket placed microphones, ambient noise levels were initially measured from a microphone placed within an empty sound-attenuated chamber. Noise levels were, on average, 33.6 dB SPL (RMS; s.d. 0.5) and 33.5 dB SPL (s.d. 0.4) before and after the microphone was positioned within the pocket of a jacket, respectively. This suggests that noise levels were not significantly attenuated by placement of the microphone within the material of the jacket. Subsequently, an animal was introduced and allowed to explore the chamber and encouraged to lick the water spout. The sound level measured approximately 5 cm away from the animal's head was, on average, 43.6 dB SPL (s.d. 3.9). After the microphone was re-positioned within a jacket worn by the same animal licking the spout, the noise level remained largely unchanged (mean: 43.5 dB SPL; s.d. 3.9). Together, these data suggest that the position of the microphone had little effect on ambient noise level measurements, even when the animal was freely moving and able to turn its head.

## 4 Discussion

The novel animal model of bilateral cochlear implantation presented here provides a new approach to study the effects of chronic intracochlear electrical stimulation on the deafened auditory system. Compared with clinical populations, animal models generally provide greater experimental control over inter-subject variables, such as age at onset of hearing loss and duration of deafness prior to implantation. Furthermore, ferrets enable functional assessments of hearing, including free-field behavior and electrophysiological measures, to be compared with morphological changes within the same implanted animals.

Our technique of bilateral cochlear implantation provides a safe and effective method for chronic, multi-channel, intracochlear electrical stimulation in the ferret. Results from electrode impedance measurements over a 3-month post-operative period suggest that the majority of electrodes remained functional for the duration of this study. Furthermore, ECAP thresholds were relatively low and remained stable throughout the assessment period. Low ECAP thresholds suggest that small current amplitudes are required for effective stimulation, which was confirmed through behavioral testing of comfort and threshold levels. Results have been presented in recent conference abstracts that suggest this model can be used to study behavioral aspects of binaural hearing with a free-field acoustic environment by connecting the intracochlear electrode arrays to clinical processors via modified stimulator-receivers worn within a custom-made jacket (Hartley et al., 2009; Isaiah et al., 2009), and will be described in full in future publications. It can also be used for electrophysiological assessments of binaural interactions within the central auditory pathway by stimulating the intracochlear arrays under direct computer control (Hartley et al., 2008).

Since intracochlear electrode impedance measurements have been shown to correlate closely with the degree of tissue response adjacent to the electrode array (Xu et al., 1997), the increases in electrode impedances within the first 3 post-operative weeks are likely to be associated with a local tissue reaction to the intracochlear prosthesis. Impedance measurements seem to remain constant, or slightly decrease in some cases, after the third post-operative week, which would suggest the level of tissue reaction to the electrode array increases up to, but not beyond, the 3rd post-operative week. Impedance measurements showed that the majority of electrodes remain intact for at least 3 months post-implantation. Indeed, within our laboratory, one ferret has been implanted with a unilateral intracochlear electrode array for >18 months, and impedance measurement from all 7 intracochlear electrodes have remained stable throughout this post-operative period (data not shown).

Unlike the current study, electrically evoked potential thresholds have been shown to increase over time in a number of other chronically implanted non-human species. In studies conducted at the Bionic Ear Institute in Melbourne (Coco et al., 2007; Xu et al., 1997), cats were deafened through aminoglycoside administration in infancy prior to cochlear implantation in adulthood. In another study from the same laboratory, profound hearing loss was induced in guinea pigs using similar deafening techniques in adulthood (Shepherd et al., 2005). In both cats and guinea pigs, it was shown that electrically evoked auditory brainstem response thresholds (EABRs) increased significantly over time for all chronically stimulated intracochlear electrodes. Compared with these animal species, intra-operative ECAP thresholds in humans are not significantly different from measurements taken many months, and often years, later (Lai et al., 2004; van Wermeskerken et al., 2006). Thus, compared with cats and guinea pigs, neuronal degeneration of the spiral ganglion following deafness may be much slower in humans (Ghorayer et al., 1980; Gleuckert et al., 2005; Hinojosa and Marion, 1983; Kerr and Schuknecht, 1968; Lindsay and Hinojosa, 1978; Linthicum and Anderson, 1991; Nadol et al., 1989, 2001; Otte et al., 1978), although this has to be qualified by the fact that animal experiments often aim at destroying all hair cells by using higher doses of aminoglycosides than is the case in humans.

The stability of ECAP thresholds measured post-operatively in the ferret appears more consistent with human data (Lai et al., 2004; van Wermeskerken et al., 2006), than the elevation in post-operative evoked thresholds previously observed in cats (Coco et al., 2007; Xu et al., 1997) and guinea pigs (Shepherd et al., 2005). It has been suggested that elevation in evoked thresholds in these species may reflect an ongoing degeneration of SGNs, shrinkage of SGN cell size or an increase in the thickness of the tissue capsule surrounding the electrode array, any of which may alter the proportion of current that is directly shunted between the stimulating electrodes (Coco et al., 2007; Shepherd et al., 2005; Shepherd and Javel, 1997). In these animal studies, aminoglycoside and loop diuretic were co-administered intravenously to induce profound hearing loss, whereas in the current study aminoglycoside was administered either directly to the scala tympani or using subcutaneous injections. Regardless of these methodological differences, Nadol and colleagues (Nadol et al., 1989) showed that humans with deafness due to aminoglycoside toxicity had the highest residual spiral ganglion cell count compared with other aetiologies of profound hearing loss. It is possible that any degeneration of SGNs associated with our deafening technique in ferrets is not great enough to alter ECAP thresholds. Alternatively, any tissue reaction associated with the chronically implanted electrode array in the ferret may not alter the proportion of current that is directly shunted between the stimulating electrodes. Within an ongoing study within our laboratory, we are investigating the effects of deafening and chronic intra-cochlear electrical stimulation on cochlear morphology in the ferret.

In patients with a CI, the microphones of the speech processor are conventionally positioned immediately antero-superior to the pinna. Until recently implants have been inserted on one side of the head only. However, many individuals with unilateral CIs experience difficulties hearing speech in background noise and localizing sounds in space (van Hoesel and Tyler, 2003). For normal listeners, abilities on these tasks are significantly improved when hearing with two ears, because of the availability of binaural localization cues. For localization in azimuth, ILDs are the most important localization cue for high-frequency sounds, whereas ITDs can be detected in the fine structure of low-frequency sounds (<1.5 kHz) and in the envelopes of high-frequency, complex sounds. Although results are variable, evidence from trials of bilateral CIs suggests that, compared with unilateral implants, bilateral devices can substantially improve sound localization (Dunn et al., 2008; Gantz et al., 2002; Grantham et al., 2007; Litovsky et al., 2004; Neuman et al., 2007; Nopp et al., 2004; Seeber et al., 2004; Tyler et al., 2002; Van Deun et al., 2010; van Hoesel, 2004; van Hoesel and Tyler, 2003) and detection of a signal against a background of interfering noise (Gantz et al., 2002; Long et al., 2006; Muller et al., 2002; van Deun et al., 2009; van Hoesel, 2004; van Hoesel and Tyler, 2003). Bilateral implants are associated with consistently good sensitivity to ILDs provided by head-mounted microphones (van Hoesel and Tyler, 2003), which is comparable with that shown by normally hearing listeners (Yost and Dye, 1988). However, using most current commercially available stimulation strategies, ITDs are generally more difficult to hear, particularly at rates above a few hundred Hz (van Hoesel and Tyler, 2003). Bilateral CIs with current commercially available stimulation strategies generally do not transmit fine-structure ITDs, due to the constant phase in the electrical pulse train (van Hoesel and Tyler, 2003).

During the development of this animal model, the jacket that the ferrets wore to carry the external speech processors and modified stimulator-receivers was designed to ensure that animals were provided with behaviourally relevant binaural cues during chronic stimulation. Our acoustical recordings suggested that positioning the microphones of the speech processors within the jacket pockets immediately behind the animal's pinna on each side of their head ensured that binaural cues were preserved, at least at the level of the microphones of the speech processors, and were very similar to the ITDs and ILDs provided by the external ear canals of adult ferrets when the animals are facing straight ahead. This is critical for the behavioural task used to assess sound localization, as the animals have to adopt this position in order to trigger the presentation of a stimulus from one of a number of possible loudspeaker locations (see Nodal et al., 2008 for details). Consequently, both ITDs and ILDs available to the CI ferrets should match those provided by the ears for corresponding source locations. In fact, we observed greater inter-animal variation in the acoustical measurements taken from the external ears, which is due to individual differences in head size (Schnupp et al., 2003). It would be straightforward to adjust the position of the jacket microphones to take these differences into account.

Our acoustical measurements also indicated that the binaural cues provided by the jacket changed dynamically with the orientation of the animal in a comparable fashion to the way the cues changed when measured from the external ear canals, although larger changes in ITDs and ILDs were seen with head orientation in external ear canal measurements than with the jacket pockets and the maximum values occurred at slightly different azimuths. These differences will be relevant only for long stimuli that are still present when the animal starts to orient towards the sound source, which occurs at about 150–250 ms after stimulus onset in normally hearing ferrets (Fig. 6, Nodal et al., 2008). Even then, given the considerable adaptive capabilities of the mature auditory localization system (Kacelnik et al., 2006), it seems likely that the animals will learn to accommodate these small differences. Importantly the initial and final bearings were well matched between the head and jacket measurements (Fig. 6), indicating that this measure of localization accuracy should produce comparable results in each case.

In the ferret, the head and body are a similar width. Therefore, although this design worked well in our chosen species, translating this design to species in which the ratio of size of the head and neck does not approach unity, such as the cat or primate, may prove less successful.

Normal hearing individuals utilize spectral cues derived from the pinna to aid sound localization. Pinna cues enable a normal hearing individual to make front-back discriminations, localize in elevation and even to localize sounds in the horizontal plane with one ear only under certain listening conditions (Wightman and Kistler, 1993). Individuals with CIs are unlikely to be able to take advantage of pinna cues, since the microphone of the speech processor is commonly positioned above the pinna, rather than within the entrance of the ear canal. Secondly, spectral cues provided by the pinna are most informative for high frequency sounds (>6 kHz) that most commercially available implant strategies rarely provide. Although our behavioural model could be used to investigate the feasibility of introducing this cue, we have not so far attempted to provide pinna cues within our current experimental design.

Prior to developing our jacket-pocket design we considered alternative methods of positioning the microphones, including speech processors secured with a surgically implanted post fixed to the skull. We are unaware of a commercially available speech processor microphone system that incorporates skull fixation suitable for human cochlear implant recipients. Therefore, arguably, a solution involving bone fixation is less clinically relevant than one that does not. Nevertheless, compared with our jackets, a head mounted system may better match the binaural cues provided by the external ear canals of an individual animal. Furthermore a skull-fixation device may improve upon the head orienting cues that are currently provided by our jackets. Conversely, when acoustic signals are presented within a free-field acoustic environment, the skull-fixation system could alter the size and/or symmetry of the head and, subsequently, change the acoustic properties of the signals at the two ears, and hence the value of the ILDs, and possibly, ITDs. Furthermore, if a head-mounting system was used to suspend the speech processors from a skull post, the speech processors would be vulnerable to striking solid objects whenever the animal negotiated its way around the behavioral test chamber or home cage. At the very least, this could generate noise artifacts and, worst still, could alter the positions of the microphones. It is also important to consider complication rates associated with microphone positioning. For many behavioral tasks, data collection can take 3 months, or longer. To ensure a period of reliable and constant data collection it is imperative to maintain the health of the animal, and integrity of the implanted electrodes, for the duration of the behavioral experiment. In animals with bilateral cochlear implants, a choice of microphone position that involves skull fixation could alter complication rates, compared with a non-surgical solution such as a jacket. A skull fixation post would necessarily involve a wound: the scalp is unable to grow over the post leaving a junction between the skin and the post. This wound would be adjacent to the lead wires that lie beneath the scalp and connect with the intracochlear electrodes. Such a wound could increase the risk of infections that are already known to be associated with cochlear implantation (e.g. wound infections, abscess formation, middle and inner ear infections and meningitis) and may compromise the fixation of the lead wires to the skull, which may, in turn, increase the risk of electrode breakage.

The model described within this manuscript includes percutaneous lead wires that can be attached to an external stimulator receiver and commercially available speech processor that the animal carries within a jacket to provide chronic intracochlear electrical stimulation within a free-field acoustic environment. Percutaneous lead wires can also be stimulated under direct computer control to deliver stimuli directly to the animal's bilateral intracochlear electrodes, rather than presenting signals via the animal's sound processors. This therefore provides an opportunity to measure ITD and ILD sensitivity independently within psychophysical or electrophysiological experiments.

Arguably, the major challenge to future CI research is to develop implants that can more closely reflect the capabilities of the human auditory system. In this respect, bilateral CIs have been trialed and evidence suggests some individuals gain significant hearing advantages from two implants. However, abilities vary considerably between individuals. It has been suggested that the substantial variation in binaural sensitivity between implanted individuals may reflect the effects of auditory experience (Seeber et al., 2004) and age at onset of hearing loss or duration of deafness prior to implantation (Dunn et al., 2008). However, the small numbers of patients with bilateral CIs tested to date and the multiple variables between individuals complicate the interpretation of these data. Animal models can largely control for these variables independently. Thus, our novel animal model in the ferret has been designed to determine how these factors influence the effectiveness of bilateral cochlear implantation. In so doing, we aim to identify patient groups that are most likely to benefit from this surgical treatment. Since our model may be used to assess behavioural measures of hearing as well as electrophysiological and histological outcome measures, we envisage that ferrets may prove valuable in the development and testing of other technological innovations in the field of cochlear implantation, such as inner ear drug delivery systems, advances in electrode technology, novel stimulation strategies, combined electro-acoustic hearing and novel rehabilitation techniques.

In summary, we have developed a novel behavioral animal model of bilateral cochlear implantation in the ferret that is both safe and effective with the aim of developing better treatments for hearing loss in humans, whilst being able to use the CI to improve our understanding of mechanisms that underlie the perception of sound.

## Figures and Tables

**Table 1:** The number of animals used for each procedure.

Procedure	Number of animals
Deafening technique, surgical approach, effectiveness of implantation and chronic stimulation	4
Depth of implantation	2

Preservation of binaural cues during chronic stimulation	
Video recording	1
External ear canal measurements	16
Jacket pocket measurements	4

Total	27
